# The Effects of Qigong on Anxiety, Depression, and Psychological Well-Being: A Systematic Review and Meta-Analysis

**DOI:** 10.1155/2013/152738

**Published:** 2013-01-14

**Authors:** Fang Wang, Jenny K. M. Man, Eun-Kyoung Othelia Lee, Taixiang Wu, Herbert Benson, Gregory L. Fricchione, Weidong Wang, Albert Yeung

**Affiliations:** ^1^Psychological Department, Guang'an Men Hospital, China Academy of Chinese Medical Sciences, Beijing 100053, China; ^2^Depression Clinical and Research Program, Massachusetts General Hospital, Boston, MA 02114, USA; ^3^Department of Social Work, University of North Carolina at Charlotte, Charlotte, NC 28223, USA; ^4^Chinese Cochrane Centre, West China Hospital, Sichuan University, Chengdu 610041, China; ^5^Benson Henry Institute Massachusetts General Hospital, Massachusetts General Hospital, Boston, MA 02114, USA

## Abstract

*Introduction*. The effect of Qigong on psychological well-being is relatively unknown. This study systematically reviewed the effects of Qigong on anxiety, depression, and psychological well-being. *Methods*. Using fifteen studies published between 2001 and 2011, a systematic review was carried out and meta-analyses were performed on studies with appropriate homogeneity. The quality of the outcome measures was also assessed. *Results*. We categorized these studies into three groups based on the type of subjects involved as follows: (1) healthy subjects, (2) subjects with chronic illnesses, and (3) subjects with depression. Based on the heterogeneity assessment of available studies, meta-analyses were conducted in three studies of patients with type II diabetes in the second group, which suggested that Qigong was effective in reducing depression (ES = −0.29; 95% CI, −0.58–0.00) and anxiety (ES = −0.37; 95% CI, −0.66–0.08), as measured by Symptom Checklist 90, and in improving psychological well-being (ES = −0.58; 95% CI, −0.91–0.25) as measured by Diabetes Specific Quality of Life Scale. Overall, the quality of research methodology of existing studies was poor. *Conclusions*. Preliminary evidence suggests that Gigong may have positive effects on psychological well-being among patients with chronic illnesses. However the published studies generally had significant methodological limitations. More high-quality studies are needed.

## 1. Introduction

The word “Qigong” is a combination of two concepts: “Qi,” the vital energy of the body, and “gong,” the skill of working of the Qi. Together, Qigong (or Chi Kung) means cultivating energy [1]. Qigong is based on Taoist philosophy and traditional Chinese medicine theories to cultivate Qi. It has a history of several thousand years, and is a highly popular practice, particularly in China, for health maintenance, healing, and increasing vitality [2]. Qigong can be divided into various categories such as static Qigong, dynamic Qigong, internal Qigong (*neiqi*), and external Qigong (*waiqi*) [3].

Qigong exercises consist of a series of orchestrated practices including body posture, movement, breathing, and meditation, all of which have been designed to enhance Qi function—that is, to draw upon natural forces to optimize and balance energy within, through the attainment of deeply focused and relaxed states [4]. An overview of the research literature pertaining to internal Qigong yields more than a dozen forms that have been studied on their effects on health outcomes, including Guo-lin, Chun-Do-Sun-Bup, Vitality or Bu Zheng Qigong, Eight Brocade, and Medical Qigong [5, 6]. As a form of gentle exercise, Qigong is composed of repetitive movements that are used for strengthening and stretching the body, increasing circulation of various fluids (blood, synovial, and lymph), enhancing balance, and building awareness of how the body moves through space [6]. From the perspective of Western philosophy and science, it could be hypothesized that Qigong, like other meditation techniques, elicits the Relaxation Response and alleviates the dysregulation of the hypothalamic-pituitary-adrenal axis [7]. The potential psychological benefits derived from the practice of Qigong may include relaxation, exposure, desensitization, deautomatization, catharsis, and counterconditioning [8].

As a form of complementary and alternative medicine, Qigong has been used to treat medical conditions such as high blood pressure [9, 10], bone loss [11], and weight-loss maintenance [12]. Short-term Qigong training appears to improve functions of the respiratory [13] and immune systems [14]. Various health claims about Qigong have been made for: hypertension [15–17], Parkinson's disease [18], Type II diabetes [19], cancer [7, 19], cardiac disease [20], pain reduction among post-surgery patients, and patients with injury, arthritis, and fibromyalgia [21, 22]. 

Several Qigong review articles have been published, which mainly focus on the effects of Qigong on specific medical conditions such as hypertension [17], cancer [19], and geriatric patients [23]. However, for health practitioners, it is still unclear whether Qigong can be recommended as an effective therapy for emotional problems and for improving psychological well-being. The purpose of this meta-analysis was to systematically review the effects of Qigong on psychological outcomes. Due to the limited number of studies in this area, we reviewed Qigong studies which reported on a relatively wide spectrum of outcomes including mood, anxiety, psychological well-being, self-efficacy, and quality of life. 

## 2. Materials and Methods

### 2.1. Data Searches and Study Selection

Since many Qigong studies were conducted in China and published only in Chinese language journals, the authors included three researchers from China and five researchers from the U.S. Electronic relevant publications from both Chinese and English databases were reviewed. Two reviewers searched and screened the titles and abstracts of the studies identified by the search against the eligibility criteria for English databases independently. One reviewer searched and screened the studies in Chinese. For potentially eligible studies, the full text publications were obtained and criteria reapplied. Disagreement was resolved by discussion. A professional librarian was consulted in our search process.

Research articles published in English on the effects of Qigong on mood and depression were identified from the following databases: from the inception to 2011 on Medline, PubMed, PsycINFO, Cochrane Reviews, Ovid, EBSCOhost, and all of the journals in the Harvard Countway Library of Medicine. Research articles published in Chinese on the effects of Qigong on mood and depression were identified from the following Chinese databases: from the inception to 2011 on CNKI, Wan Fang Med Online, and VMIS. For English databases, the key words used included a combination of MeSH and free text terms: “Qigong/Qi Gong/Gong, Qi/Ch'i Kung/Kung, Ch'i”, “mood,” “depression,” “anxiety,” “emotional well-being,” and “psychological well-being” as main subject headings, text words in titles, and abstracts. For Chinese databases, the key words used included equivalent Chinese terms as main subject headings, text words in titles, and abstracts. 

**Figure 1:**
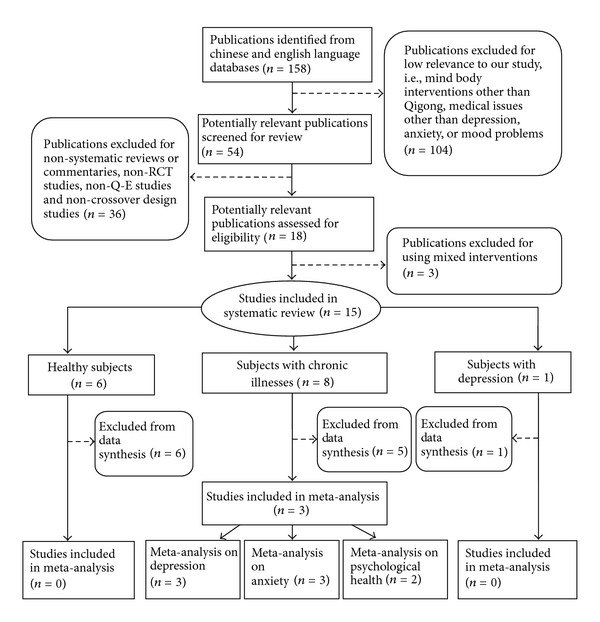
Flow chart of the study selection process.

According to the selection criteria, interventions were restricted to Qigong. Other psychological interventions such as yoga and meditation were excluded; mixed interventions (e.g., acupuncture and Qigong in combination) were excluded (as described in Figure 1). The primary outcomes evaluated were psychological, with particular emphasis on mood, anxiety, depression, self-efficacy, and quality of life. 

To be included in the meta-analyses, studies needed to have either a randomized controlled trial (RCT) or quasi-experimental (Q-E) design. The process of study selection was described in Figure 1. A study was operationally defined as RCT in this paper if the allocation of participants to treatment and comparison groups was reported to be randomized. If allocation of participants was done through a systematic sequence (e.g., alternate days of the week) without randomization, the study was operationally defined as having a Q-E design in this paper. Studies that did not use any type of comparison group, or did not report any comparison results between groups, or used mixed interventions were excluded. Duplicate publications were also excluded. Titles and abstracts gathered from the databases were first reviewed for relevance to this paper. The full text of papers that met the inclusion criteria were then obtained, and findings were summarized.

### 2.2. Data Extraction and Quality Assessment

We assessed the characteristics of the original research and extracted data accordingly. Some basic information was collected based on date of publication, study sites, language of study, and clinical domains (see Table 1). The methodological quality of RCTs was evaluated based on six criteria: adequate sequence generation, allocation concealment, blinding which including the blindness adopted during the conduct and analysis of the studies, completeness of outcome data, selective reporting, and other potential biases, for which the compliance assessment, similarity of comparison groups at baseline and appropriateness of the statistical analyses should be assessed [24, 25] (Table 2).

Findings of 15 studies were tabulated regarding sample characteristics (i.e., total sample size, age, gender, number of participants in Qigong group), duration, intervention style, design of control measures being used, and main outcomes (Table 3). Two reviewers extracted data and assessed the quality of each study independently. Strength of interreviewer agreement was expressed using Cohen *k* coefficient [26]. Disagreement was initially resolved by discussion. When related data were not provided in articles, trial authors were contacted through e-mail or phone.

#### 2.2.1. Assessment of Heterogeneity

If substantial clinical, methodological, or statistical heterogeneity existed, study results were not combined by means of meta-analysis. Clinical heterogeneity usually came from patients' characteristics (age, gender, etc.). Methodological heterogeneity refers to differences between studies in terms of methodological factors, such as sequence generation and concealment of allocation [24]. If the studies did not have these heterogeneities, we performed a meta-analysis and determined whether they showed statistical heterogeneity by visually inspecting the forest plots and by using a standard *χ* ^2^-test with a significance level of *α* = 0.1, given the low power of such tests. Statistical heterogeneity was specifically examined with *I* ^2^ [25], where *I* ^2^ values of 50% or more indicate a substantial level of heterogeneity [27]. When heterogeneity was found, we attempted to determine potential reasons for it by examining individual study characteristics. 

#### 2.2.2. Assessment of Reporting Biases

Because all 15 studies we reviewed had small samples, funnel plots were used in an exploratory analysis to assess the potential existence of small study bias if 9 or more studies were included in one meta-analysis. If less than 9 studies were included in the meta-analysis, we considered that a potential risk of selective reporting existed [24]. 

#### 2.2.3. Data Statistical Analysis and Quality Assessments of Outcome Measures

Since all outcomes were continuous variables, if the same measurement was used across studies, effect size (ES) was determined by calculating the mean difference between groups. If the same underlying concept was measured but different outcome measurements were used, ES was determined by calculating the standardized mean difference between groups. 

Because of the different trials implemented various styles of Qigong, if any trials with three or more treatment arms were identified, we made two assumptions for the analysis. Firstly, if the trial was comparing two or more styles of Qigong versus control, then the data for those Qigong arms were combined to give one comparison of Qigong intervention versus control for that trial.

Secondly, if the trial was comparing Qigong versus two or more controls, then the data for those control arms were kept separate, and the data for that trial were included in the appropriate control categories. 

Overall outcome was assessed by pooling the ES of each study. In view of the heterogeneity, random-effects model was used for pooling. All analyses were conducted using Review Manager 5 (Version 5.0; The Chinese Cochrane Centre, The Cochrane Collaboration; Chengdu, China). We assessed the quality of the outcome of measures using GRADE profiler version 3.

## 3. Results

### 3.1. Study Description

Fifteen studies published between 2001 and 2011 were included in this systematic review. Of these, 6 were published in Chinese and identified from Chinese databases [28–33] and 9 were published in English and identified from English databases [15, 34–41]. Disagreement for articles included was on 5 of 20. They were excluded after discussion. 

 Only one of these studies was conducted in the United States; the majority (*n* = 9) of the remaining studies were conducted in China, including Hong Kong. In six studies, effects of Qigong interventions were examined in healthy adults without any specific medical conditions. The majority of the studies, however, targeted individuals with a variety of chronic conditions, including diabetes (*n* = 4), depression (*n* = 1), cancer (*n* = 1), and hypertension (*n* = 2) (Table 1).

Table 2 presents the methodological quality of the 15 studies reviewed. All studies, with the exception of one Q-E study [15], were RCTs. Ten studies used a two-arm design with one intervention and one control group, and the remaining four adopted a three-arm design which used either a different type of Qigong [33] or a psychoeducational group [28, 36, 41] as the second comparison group. The interrater agreement as measured by kappa (*κ*) was 0.901 (*P* < 0.0005). 

Seven studies described the randomization process. One study reported that the randomization was performed by a statistician who had prepared a randomization list before the study started [34]. Four studies reported that the randomization was performed through the use of computer-generated numbers [31, 32, 35, 38]. Two studies used a random-number table [28, 37]. The other seven studies did not clearly report the process of randomization [29, 30, 33, 36, 39–41]. One study allocated the participants according to their place of residence [15], which cannot be considered a sufficient randomization. Two studies specified allocation concealment by using the allocation sequences sealed in opaque envelopes [31, 32]. 

Blinding was described in only three studies. One study adopted a single blind run-in period [34]. Another study reported that the treatment order was randomly determined and subjects did not know their treatment [37]. The other study adopted a double-blind method as to group assignment of treatment procedure [40]. Blinding the participants to the allocation was not adopted in one study while the other blindness such as study analysis was not described clearly [38]. The majority of studies addressed incomplete outcome data. Three studies used intention-to-treat analyses [31, 34, 38]. Eight studies reported the number of drop-outs and related reasons [15, 28, 34–38, 40]. Three studies reported the number of drop-outs, but did not explain the reasons for drop-outs [31, 32, 39]. Two studies described the periods of follow-up [15, 40]. Through careful reading of the study and contacting the study authors for additional information, we tried to examine whether there was selective reporting of outcomes. Two studies reported all outcome measurements [30, 32]. One study did not address all of the outcomes [31]. For the majority of the studies, the existence of selective reporting could not be determined due to inadequate information.

Five studies described the methods to evaluate the adherence of patients to intervention [30, 34, 35, 38, 39]. Comprehensive comparisons of demographic and baseline information were presented in eight studies [28, 31, 32, 34, 35, 38, 40, 41], two of which reported that some demographic characteristics were unbalanced among comparison groups at baseline [34, 40]. The statistical methods in all of the included studies were considered appropriate for the analyses performed.

Table 3 summarized the 15 studies with regard to effects of Qigong on psychological well-being outcomes. The study sample sizes ranged from 20 to 162, with a total of 1154 research participants. Among them, 593 subjects received Qigong intervention. All studies recruited participants aged 18 years and up with the majority in their middle adulthood. Two studies targeted participants aged 65 and older [40, 41], and one study recruited young adults in college settings [33]. Most studies include mixed gender groups, though one study included males only [37].

The durations of the interventions ranged from 70 minutes to 4 months. Interventions of 3-4 months' duration appeared to be the norm for demonstrating changes while maximizing study enrollment and adherence. Among the Qigong intervention studies, the most popular form was the “Eight Section Brocade Exercise.” During and outside of group practice sessions, peer learning and discussions to facilitate social interaction and mutual support were encouraged since these may be important therapeutic ingredients. In most of the studies, control groups received treatment as usual and routine medical check-up. Three studies utilized a waitlist as the control group [15, 35, 39]. 

While all included studies reported on psychological outcomes, only the study by Tsang et al. targeted participants with a psychiatric disorder [40]. The remaining studied either healthy subjects or subjects with chronic medical conditions, and examined psychological factors as secondary goals of the study. 

The most frequently reported psychological benefits were decreased depressive symptoms and improved mood, reported in seven studies [28–31, 36–38], as evidenced by scores on depression scales (e.g., Hamilton Depression Severity Index-17, Self-Rating Depression Scale, Center for Epidemiological Studies Depression Scale, etc.). Depression was shown to improve significantly in studies comparing Qigong to an inactive control, newspaper reading [40], usual care, psychosocial support, or stretching/education controls [30]. General measures of mood (e.g., Profile of Mood States) improved significantly for those practicing Qigong compared to a wait-list control group [15]. In two studies, depressive symptoms improved, but the change was not statistically significant, for both Qigong and for exercise comparison groups [34, 41]. 

Participants in the intervention groups also demonstrated reduced anxiety [29–31, 37, 41], as assessed by scales such as the Self-Rating Anxiety Scale. Anxiety decreased significantly for participants practicing Qigong compared to an active exercise group [15, 34]. 

Three studies reported statistically significant improvements in somatic symptoms among the intervention group as evidenced by scales such as the Symptom Checklist-90 and Somatization Scale [28]. In these studies, participants also reported lower perceived stress and intensity of pain compared with the control group. 

Some studies employed measures of physical health and biomarkers, including blood pressure [15], cholesterol levels [30, 31], fasting blood sugar [29, 30, 32], and triglycerides [29, 30]. In one study examining biomarkers related to stress response, norepinephrine, epinephrine, and blood cortisol levels were significantly decreased in response to Qigong compared to a wait-list control group [15].

Improvement of overall quality of life (QOL) was the second most frequently mentioned benefit reported in six studies [28, 29, 31, 34, 40, 41]. In studies with heterogeneous participants (including healthy adults, patients with cancer, post-stroke, arthritis, etc.), at least one of the components of QOL was reported to be significantly improved by Qigong compared to newspaper reading [40] or traditional remedial rehabilitation [41]. In one study, Qigong showed improvements in QOL compared to an exercise intervention, but the results did not reach statistical significance [34]. With a few exceptions, the majority of studies indicate that Qigong holds great potential for improving QOL in both healthy and chronically ill patients.

Self-efficacy was generally assessed in the RCTs as a secondary outcome related to the problem area under investigation (e.g., efficacy to manage a disease or pain symptom, or in the case of falls among the elderly, feeling more confident that one will not fall). The perceived ability to handle stress or novel experiences [15, 40] and exercise self-efficacy [15] were found to be enhanced in the Qigong intervention groups relative to control groups.

### 3.2. Meta Analyses for Three Subgroups

We categorized the studies into three groups based on the type of subjects for further analysis as follows: (1) healthy subjects, (2) subjects with chronic illnesses, and (3) subjects with depression. Only one RCT recruited subjects with depression [40] and therefore no meta-analysis was needed for this group. Six RCTs were included in the group of studies with healthy subjects [29, 33, 35–37, 39]. Meta-analysis was not performed in this group. One study recruited only male participants, which made it hard to compare to other studies [37]. Another study used a crossover design with each participant serving as his or her own control without a separate comparison group [39]. The remaining four studies used different groups as controls, including a lecture [36], Tai Chi and fitness Yangko [29], a waitlist [35], and an unclearly described control [33].

Eight RCTs were included in the group of studies of patients with chronic illnesses [5, 28, 30–32, 34, 38, 41]. Five studies were excluded from the meta-analysis. One study was quasi-experimental [15], and another study had a high dropout rate (32% in the intervention group and 35% in the control) [38]. The other three studies were excluded since they used a different control group than the three RCTs included in meta-analysis, which used treatment as usual and no Qigong intervention as control. Three of the excluded studies used the following control conditions—conventional exercise [34], traditional remedial rehabilitation under the supervision of qualified professionals [41], and health education [28].

After assessment of heterogeneity and consideration of the choices of varying control groups used in different studies, meta-analysis of outcomes related to depression measured by Symptom Checklist 90 (SCL-90) were performed on the remaining three RCTs of patients with type II diabetes [30–32]. Baseline characteristics were reasonably well balanced between the Qigong group and the control group for the three trials. At endpoint, there were a significant differences between the two groups on obsessive-compulsive, depression, anxiety and anger-hostility in Wang's study, on somatization in Lin's study, and on phobic anxiety in Zhang's study (*P* < 0.05). Results of the individual trials for SCL-90 are presented in Table 4.

**Figure 2:**
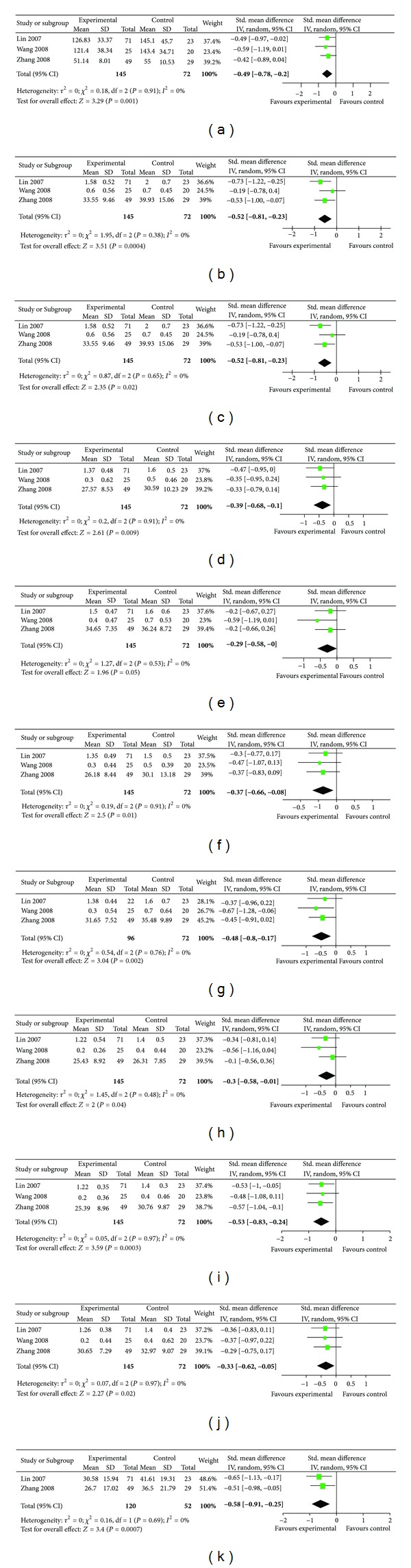
Effects of Qigong on symptoms of SCL-90 and psychological health of DSQL in subjects with chronic illnesses.

We found significant differences between groups (ES = −0.29, 95% CI, −0.58–0.00), with *I* ^2^ = 0% (Figure 2(e)). Meta-analysis of outcomes related to anxiety were also performed in the same three studies [30–32]. We found significant differences between groups (ES = −0.37; 95% CI, −0.66–0.08), with *I* ^2^ = 0% (Figure 2(f)). 

Besides depression and anxiety, meta-analysis of other symptoms of SCL-90 were also performed in the same three studies. We found significant differences between groups in total SCL-90 score (ES = −0.49; 95% CI, − 0.78 to −0.20), somatization (ES = − 0.52; 95% CI, − 0.81  to −0.23), obsessive-compulsive (ES = −0.35; 95% CI, − 0.64 to  −0.06), interpersonal sensitivity (ES = −0.39; 95% CI, −0.68 to −0.10), anger-hostility (ES = −0.48; 95% CI, −0.80 to −0.17), phobic anxiety (ES = −0.30; 95% CI, −0.58 to −0.01), psychotism (ES = −0.53; 95% CI, −0.83 to −0.24) and paranoid ideation (ES = −0.33; 95% CI, −0.62 to −0.05). All the above outcomes were with *I* ^2^ = 0% (Figures 2(a)–2(d), 2(g)–2(j)). 

Two RCTs were included in the meta-analysis of psychological health measured by Diabetes Specific Quality of Life Scale (DSQL) [29, 36]. Baseline characteristics were reasonably well balanced between the Qigong group and the control group for the two trials. At endpoint, there was a significant difference between the two groups on psychological health (*P* < 0.05). Results of the individual trials for DSQL are presented in Table 4.

We also found significant differences between groups (ES = −0.58, 95% CI, −0.91−0.25), with *I* ^2^ = 0% (Figure 2(k)). Data synthesis showed that Qigong was effective in reducing depression and anxiety and improving psychological well-being among subjects with type II diabetes. Yet the quality of the outcomes measures used in these studies was low (Table 5).

## 4. Discussion

The studies in this paper demonstrated that Qigong may have beneficial effects for a variety of populations on a range of psychological well-being measures, including mood, anxiety, depression, general stress management, quality of life, and exercise self-efficacy. The movements of Qigong is relatively easy to learn, when compared to other mind body traditions [2, 4]. Hence, people from diverse backgrounds practice Qigong for a variety of reasons, including exercise, recreation, well-being, self-healing, meditation, self-cultivation, and training for martial arts. We see a great potential for Qigong to be integrated for the prevention and treatment of various chronic illnesses, including psychiatric disorders. 

This systematic review highlights the mood and psychological effects of Qigong in addition to its physical effects. The outcomes of the three selected studies showed improvements in psychological well-being, especially when the control intervention does not include active interventions such as exercise. These studies used SCL-90 to measure the pre- and post-outcomes related to Qigong intervention. While SCL-90 is a widely used and well validated measure for psychological outcomes, it is important to point out that it does not provide information on clinical diagnoses of anxiety of depressive disorders. Due to the small number of studies available in this area, and the diverse outcomes used, we limited meta-analysis on patients with diabetes. With more relevant studies in the future, it will be informative to review separately, the anxiety and depressive outcomes among healthy subjects, patients with specific chronic illness (e.g., fibromyalgia, tension headache, etc.), and for patients with specific psychiatric disorders (e.g., generalized anxiety disorder, panic disorder, major depressive disorder, etc.). 

Qigong practice usually involves doing Qigong (movements with breathing exercises and visualization), plus peer learning, social support, and positive expectation. All these could have beneficial effects to psychological well being and so all these are encouraged in Qigong practice. We have acknowledged that the outcomes of studying such Qigong practices will not provide us with the information on the question whether Qigong (movements with breathing exercise and visualization) alone is beneficial to psychological well being. Positive expectations or social interactions may add to effects related to the Qigong intervention, to form a multi-component mind-body practices instead of a single (Qigong) intervention.

In this paper, we included studies both from the Chinese and in English databases. We consider this approach a strength as many Qigong studies continue to be originated in China and published in Chinese language. While only one researcher performed literature search in Chinese which may lead to some biases, early Qigong research findings published before 2003 (in English), respectively, 2000 (in Chinese) have not been considered. This approach has substantially limited the literature base for the present review and consequently also its findings. The findings of this study should be interpreted in light of the methodological limitations of the studies reviewed. In both of the English and Chinese studies included in the review, most of them used treatment as usual (and one used a waitlist) for the control group. This may lead to bias since positive outcomes from the study could be due to positive expectations or social interactions rather than to the Qigong intervention. A sham treatment which offers social interaction and positive expectations from receiving an intervention could be a better control for these studies. It will also be important in future studies to control for what has been called the frustrebo effect (i.e., negative effects emanating from subject frustration in not receiving the kind of intervention they feel they need) [42].

The majority of these RCTs were pilot studies on patients with chronic illnesses conducted to collect preliminary data on the efficacy of a group intervention to estimate the effect size needed for a larger, more definitive study. While the studies provided valuable data regarding feasibility and clinical efficacy, the use of a small sample could lead to instability of the outcomes, making it harder to generalize to other populations. In addition, many studies used inadequate blinding of the intervention, which could lead to more favorable responses among the Qigong intervention groups. Most of the cited studies did not provide data on whether participants continued to practice Qigong after the intervention period. Subsequently, long-term psychological effects of Qigong are unclear. 

Generally, Qigong practices are considered safe, and there have been few published adverse events [2, 4]. While Qigong induced psychosis has been reported The prevalence has been very low [43, 44]. However, there have been no systematic reviews of its risks either. The potential risks of this practice may have been underestimated, reflecting underreporting of adverse events in studies and in practice. In sum, preliminary evidence from the current literature suggests that Qigong may have positive psychological effects for the chronically ill individuals with symptoms of depression and/or anxiety. However, the studies reviewed generally had significant methodological limitations. Future RCTs with rigorous research design based upon the CONSORT statements [45] are needed to establish the efficacy of Qigong in improving psychological well-being and its potential to be used as interventions for populations with various clinical conditions. 

## Figures and Tables

**Table 1:** Summary of Qigong studies reviewed.

	No. of studies	Study ID no.
Date of publication		
2001–2005	4	16, 32, 35, 41
2006–2011	13	28, 29, 30, 31, 33, 34, 36, 37, 38, 39, 40
Study sites		
US	1	33
China and Hong Kong	11	28, 29, 30, 31, 32, 36, 37, 40, 41
Others (Sweden, Australia, Korea)	5	16, 34, 35, 38, 39
Language of study		
Chinese	6	28, 29, 30, 31, 36, 37
English	11	16, 32, 33, 34, 35, 38, 39, 40, 41
Clinical domains		
Chronic physical illnesses	1	41
Cancer	1	38
Depression	1	40
Type II diabetes	4	28, 30, 31, 36
Hypertension	2	16, 32
No medical condition	8	29, 33, 34, 35, 37, 39

**Table 2:** Methodological quality of Qigong studies reviewed.

Lead author	Adequate sequence generation	Allocation concealment	Blinding	Completeness of outcome data	Selective reporting	Other potential biases
Cheung [34]	Y	Unclear	Y	Y	Unclear	Y
Griffith [35]	Y	Unclear	Unclear	Y	Unclear	Unclear
Huo [28]	Y	Unclear	Unclear	Y	Unclear	Unclear
Jin [29]	Unclear	Unclear	Unclear	Unclear	Unclear	Unclear
Johansson [36]	Unclear	Unclear	Unclear	Y	Unclear	Unclear
Lee [16]	N	Unclear	Unclear	Y	Unclear	Unclear
Lee [37]	Y	Unclear	Y	Y	Unclear	Unclear
Lin [32]	Y	Y	Unclear	N	N	Unclear
Liu [33]	Unclear	Unclear	Unclear	Unclear	Unclear	Unclear
Oh [38]	Y	Unclear	Unclear	Y	Unclear	Unclear
Skoglund [39]	Unclear	Unclear	Unclear	N	Unclear	Unclear
Tsang [40]	Unclear	Unclear	Y	Y	Unclear	Y
Tsang [41]	Unclear	Unclear	Unclear	Unclear	Unclear	Unclear
Wang [30]	Unclear	Unclear	Unclear	Unclear	N	Unclear
Zhang [31]	Y	Y	Unclear	N	Y	Unclear

**Table 3:** Summary of Qigong studies reviewed 2.

Group	Lead author	Sample size	Age (y)	Intervention (Qigong) and controls	Duration	Psychological well-being related measures	Psychological well-being related outcomes
	Griffith [35]	*N*1 = 50 11 males, 39 females *N*2 = 25	Mean age 51	G1 Qigong practice G2 Wait-list (control)	6 weeks	Perceived Stress Scale, SF-36	Qigong improved perceived stress and social interaction subscale of the SF-36 versus control.
	Jin [29]	*N*1 = 105 *N*2 = 35	60–69	G1 Health qigong G2 Tai Chi (control) G3 Fitness Yangko (control)	12 weeks	SDS, SAS, SRHMS	SAS reduced significantly in G1, G1, G3. SDS reduced significantly in G1, G3.
Healthy subjects	Johansson [36]	*N*1 = 59 8 males, 51 females *N*2 = 28	Mean age 50.8	G1 Qigong (Jichu Gong) G2 Lecture (control)	4 days intensive	POMS, STAI	POMS-depression, anger, fatigue, STAI-state anxiety scores reduced significantly in G1.
	Lee [37]	*N*1 = 20 Male only *N*2 = 10	Mean age 26	G1 Qigong (Korean Qi-therapy) G2 Placebo (control)	70 minutes	The Spielberger Anxiety Inventory-State, MT	Qigong improved anxiety versus control.
	Liu [33]	*N*1 = 100 *N*2 = 50	College students	G1 eight-section Brocade qigong G2 unclear (control)	12 weeks	SCL-90	Qigong improved SOM, O-C, I-S and PAR versus control. SOM, O-C, DEP, ANX, HOS, other symptoms and mean score reduced significantly in G1.
	Skoglund [39]	*N*1 = 42 9 males, 33 females *N*2 = 21	42–54 Mean age 48	G1 Qigong (Shuxingpingxegong) G2 Wait-list (control)	6 weeks	Questionnaire about health state, health grading and grading of stress, a visual analogue scale (similar to a thermometer), SF12 (a short version of SF-36)	The health related quality of life was improved significantly after Qigong.

	Cheung [34]	*N*1 = 91 37 males, 51 females (88 subjects completed) *N*2 = 47	Mean age 54 (88 subjects completed)	G1 Goulin qigong G2 Conventional exercise (control)	16 weeks	SF-36, BAI, BDI	No significant difference found between the two groups.
	Huo [28]	*N*1 = 80 28 males, 41 females (69 subjects completed) *N*2 = 40	Mean age 64.2 (69 subjects completed)	G1 Eight-Section Brocade qigong G2 Health education (control)	12 weeks	SDS, DMQLS	Qigong improved SDS, total score, physiological dimension, satisfactory dimension of quality of life versus control. SDS, total score, physiological dimension, satisfactory dimension of QOL reduced significantly in G1. Satisfactory dimension of QOL reduced significantly in G2.
	Lee [16]	*N*1 = 46 *N*2 = 22	40–65 Mean age 53 (36 subjects completed)	G1 Qigong exercise G2 Wait-list (control)	8 weeks	The general self-efficacy scale, Exercise self-efficacy, the scale to measure the effect of emotion on exercise	Self-efficacy and other cognitive perceptual efficacy variables improved significantly in G1.
Subjects with chronic illnesses	Lin [32]	*N*1 = 108 28 males, 80 females *N*2 = 81	37–70 Mean age 58 (94 subjects completed)	G1 eight-section Brocade qigong G2 eight-section Brocade and static qigong G3 Static qigong G4 Treatment as usual (control)	4 months	MMPI, SCL-90, DSQL	MMPI: SI, difference of Pd in G3, difference of Pd, Pt and Sc in G2 were improved versus control. Pd, Pt and Sc reduced significantly in G2. Hy, Pd and Pa reduced significantly in G3; Pd, Sc increased significantly in G4. SCL-90: SOM in G1 were improved versus control. SOM, O-C, DEP and GSI reduced significantly in G1. O-C, HOS and GSI reduced significantly in G2. DSQL: total score, physical score, psychological score, social score and treatment score in G2, total score, social score and treatment score in G3, treatment score in G1 were improved versus control. Difference of total score, physical score and social score in G1, G2, G3, difference of psychological score and treatment score in G2 were improved versus control. Total score, physical score and psychological score reduced significantly in G2, G3. Social score and treatment score reduced significantly in G2. Total score, physical score and social score increased significantly in G4.
	Oh [38]	*N*1 = 162 69 males, 93 females *N*2 = 81	31–86 Mean age 60	G1 modified qigong G2 group therapy (control)	10 weeks	Functional Assessment of Cancer Therapy—General (FACT-G), the Functional Assessment of Cancer Therapy—Fatigue (FACT-F), POM.	Qigong improved QOL, fatigue and mood disturbance versus control.
	Tsang [41]	*N*1 = 50 26 males, 24 females *N*2 = 25	≥65 Mean age 75	G1 eight-section Brocades qigong G2 traditional remedial rehabilitation (control)	12 weeks	GDS, Perceived Benefit Questionnaire, WHOQOL-BREF[HK], ASSEI	Physical health, activity level, psychological health, social relationship, and health in general improved significantly in G1.
	Wang [30]	*N*1= 54 23 males, 31 females *N*2 = 27	41–70 Mean age 58.8	G1 eight-section Brocade qigong G2 treatment as usual (control)	4 months	SCL-90	Qigong improved O-C, DEP, ANX and HOS versus control 2 months later. HOS reduced significantly in G1 4 months later.
	Zhang [31]	*N*1 = 90 29 males, 61 females *N*2 = 60	37–69 Mean age 57.8 (78 subjects completed)	G1 eight-section Brocade and relaxation qigong G2 Liuzijue and relaxation qigong G3 Treatment as usual (control)	4 months	SCL-90, DSQL	SCL-90: SOM and PSY in G2 were improved versus control. Difference of SOM and PSY in G1, G2 were improved versus control. GSI, mean score and SOM reduced significantly in G1, G2. PST and DEP reduced significantly in G1. I-S, HOS and PSY reduced significantly in G2. QOL: physical score in G1, G2 were improved versus control. Psychological and social score reduced significantly in G2. Social score reduced significantly in G3.

Subjects with depression	Tsang [40]	*N*1 = 97 *N*2 = 41 16 males, 66 females (82 subjects completed)	≥65 Mean age 82 (82 subjects completed)	G1 Qigong practice G2 Newspaper reading (control)	16 weeks	GDS, CGSS, PWI, GHQ-12, ASSEI, Perceived Benefit Questionnaire	Qigong improved mood, self-efficacy, personal well-being, physical and social domains of self-concept versus control 8 weeks later. 16 weeks later, the improvement generalized to the daily task domain of the self-concept.

Total Sample Size (*N*1); number of participants recruited in Qigong group (*N*2); Group 1 (G1); Group 2 (G2); Group 3 (G3); Group 4 (G4); Health Status Survey Short Form (SF-36); Self-rating depression scale (SDS); Self-rating anxiety scale (SAS); Self-rated Health Measurement Scale (SRHMS); Profile of Mood States (POMS); the State and Trait Anxiety Inventory (STAI); Tuchman's mood thermometer (MT); Symptom Checklist 90 (SCL-90); Somatization (SOM); Obsessive-compulsive (O-C); Interpersonal sensitivity (I-S); Paranoid Ideation (PAR); Depression (DEP); Anxiety (ANX); Hostility (HOS); Beck Anxiety Inventory (BAI); Beck Depression Inventory (BDI); Diabetes Specific Quality of Life Scale (DMQLS); Minnesota MultiPhasic Personality Inventory (MMPI); Diabetes Specific Quality of Life Scale (DSQL); Social introversion (SI); Psychopathic deviate (Pd); Psychasthenia (Pt); Schizophrenia (Sc); Hysteria (Hy); Paranoia (Pa); Global Severity Index (GSI); The Geriatric Depression Scale (GDS); the Hong Kong Chinese Version World Health Organization Quality of Life: Abbreviated Version (WHOQOL-BREF[HK]); Self-concept Scale (ASSEI); Psychoticism (PSY); Positive Symptom Total (PST); the Chinese General Self-efficacy Scale (CGSS); Personal Well-being Index (PWI); General Health Questionnaire-12 (GHQ-12).

**Table 4:** Results of trials included in meta-analysis on symptoms of SCL-90 and psychological health of DSQL in subjects with chronic illnesses.

	Wang et al. 2008 [30]				Lin 2007 [32]				Zhang 2008 [31]			
	Baseline		Endpoint		Baseline		Endpoint		Baseline		Endpoint	
	G1 (*n* = 25)	G2 (*n* = 20)	G1 (*n* = 25)	G2 (*n* = 20)	G1 (*n* = 71)	G2 (*n* = 23)	G1 (*n* = 71)	G2 (*n* = 23)	G1 (*n* = 49)	G2 (*n* = 29)	G1 (*n* = 49)	G2 (*n* = 29)
SCL-90												
Total score	131.0 (32.2)	142.3 (50.1)	121.4 (38.3)	143.4 (34.7)	139.2 (43.4)	145.1 (45.7)	126.8 (33.4)	145.1 (45.7)	50.7 (7.7)	48.6 (7.2)	51.1 (8.0)	55.0 (10.5)
Somatization	0.7 (0.5)	0.7 (0.8)	0.6 (0.6)	0.7 (0.5)	1.7 (0.7)	2.0 (0.7)	1.6 (0.5)*	2.0 (0.7)	41.3 (12.4)	40.8 (14.4)	33.6 (9.5)	39.9 (15.1)
Obsessive-compulsive	0.7 (0.4)	0.7 (0.7)	0.5 (0.5)*	0.8 (0.5)	1.9 (0.6)	1.8 (0.5)	1.7 (0.5)	1.8 (0.5)	38.9 (11.5)	36.5 (11.6)	35.9 (8.7)	38.5 (10.4)
Interpersonal sensitivity	0.4 (0.4)	0.4 (0.6)	0.3 (0.6)	0.5 (0.5)	1.5 (0.6)	1.6 (0.5)	1.4 (0.5)	1.6 (0.5)	31.7 (14.5)	29.8 (10.3)	27.6 (8.6)	30.6 (10.2)
Depression	0.5 (0.4)	0.4 (0.6)	0.4 (0.5)*	0.7 (0.5)	1.7 (0.6)	1.6 (0.6)	1.5 (0.5)	1.6 (0.6)	38.6 (12.0)	34.7 (8.2)	34.7 (7.4)	36.2 (8.7)
Anxiety	0.3 (0.4)	0.3 (0.7)	0.3 (0.4)*	0.5 (0.4)	1.4 (0.6)	1.5 (0.5)	1.4 (0.5)	1.5 (0.5)	33.0 (16.1)	29.7 (13.6)	26.2 (8.4)	30.1 (13.2)
Anger-hostility	0.6 (0.5)	0.7 (0.7)	0.3 (0.5)*	0.7 (0.6)	1.5 (0.5)	1.6 (0.7)	1.4 (0.4)	1.6 (0.7)	35.6 (10.2)	36.9 (14.7)	31.7 (7.5)	35.5 (9.9)
Phobic anxiety	0.2 (0.2)	0.5 (0.7)	0.2 (0.3)	0.4 (0.4)	1.3 (0.6)	1.4 (0.5)	1.2 (0.5)	1.4 (0.5)	29.3 (14.7)	26.8 (9.1)	25.4 (8.9)*	26.3 (7.9)
Psychotism	0.3 (0.4)	0.5 (0.6)	0.2 (0.4)	0.4 (0.5)	1.3 (0.5)	1.4 (0.3)	1.2 (0.4)	1.4 (0.3)	31.2 (13.8)	28.1 (8.7)	25.4 (9.0)	30.8 (9.9)
Paranoid ideation	0.3 (0.4)	0.4 (0.5)	0.2 (0.4)	0.4 (0.6)	1.3 (0.5)	1.4 (0.4)	1.3 (0.4)	1.4 (0.4)	31.8 (9.6)	29.1 (9.1)	30.7 (7.3)	33.0 (9.1)
DSQL												
Psychological health					35.0 (19.0)	35.2 (17.2)	30.6 (15.9)*	41.6 (19.3)	32.7 (19.0)	36.4 (19.3)	26.7 (17.0)*	36.5 (21.8)

Note: Qigong group (G1); control group (G2). Outcomes were reported by mean (SD). *The difference between the scores of the two groups was significant (*P* < 0.05).

**Table 5:** Quality assessment of outcome measures in subjects with chronic illnesses.

Quality assessment							Summary of findings					
							No. of patients		Effect		Quality	Importance
No. of studies	Design	Limitations	Inconsistency	Indirectness	Imprecision	Other considerations	Qigong	Control	Relative (95% CI)	Absolute	
Total score (measured with: total score of Symptom Checklist 90 at end of treatment; range of scores: 0–450; better indicated by less)												

3	Randomised trial	Serious^1^	No serious inconsistency	No serious indirectness	No serious imprecision	Reporting bias^2^	145	72	—	MD 0 (−0.78 to −0.2)	*⨁⨁*○○ Low	Important^3^

Somatization (measured with: somatization score of Symptom Checklist 90 at end of treatment; range of scores: 0–48; better indicated by less)												

3	Randomised trial	Serious^1^	No serious inconsistency	No serious indirectness	No serious imprecision	Reporting bias^2^	145	72	—	MD 0 (−0.81 to −0.23)	*⨁⨁*○○ Low	Important^3^

Obsessive-compulsive (measured with: obsessive-complusive score of Symptom Checklist 90 at end of treatment; range of scores: 0–40; better indicated by less)												

3	Randomised trial	Serious^1^	No serious inconsistency	No serious indirectness	No serious imprecision	Reporting bias^2^	145	72	—	MD 0 (−0.64 to −0.06)	*⨁⨁*○○ Low	Important^3^

Interpersonal sensitivity (measured with: interpersonal sensitivity score of Symptom Checklist 90 at end of treatment; range of scores: 0–36; better indicated by less)												

3	Randomised trial	Serious^1^	No serious inconsistency	No serious indirectness	No serious imprecision	Reporting bias^2^	145	72	—	MD 0 (−0.68 to −0.1)	*⨁⨁*○○ Low	Important^3^

Depression (measured with: depression score of Symptom Checklist 90 at end of treatment; range of scores: 0–52; better indicated by less)												

3	Randomised trial	Serious^1^	No serious inconsistency	No serious indirectness	No serious imprecision	Reporting bias^2^	145	72	—	MD 0 (−0.58 to 0)	*⨁⨁*○○ Low	Important^3^

Anxiety (measured with: anxiety score of Symptom Checklist 90 at end of treatment; range of scores: 0–40; better indicated by less)												

3	Randomised trial	Serious^1^	No serious inconsistency	No serious indirectness	No serious imprecision	Reporting bias^2^	145	72	—	MD 0 (−0.66 to −0.08)	*⨁⨁*○○ Low	Important^3^

Anger-hostility (measured with: anger-hostility score of Symptom Checklist 90 at end of treatment; range of scores: 0–24; better indicated by less)												

3	Randomised trial	Serious^1^	No serious inconsistency	No serious indirectness	No serious imprecision	Reporting bias^2^	145	72	—	MD 0 (−0.8 to −0.17)	*⨁⨁*○○ Low	Important^3^

Phobic anxiety (measured with: phobic anxiety score of Symptom Checklist 90 at end of treatment; range of scores: 0–28; better indicated by less)												

3	Randomised trial	Serious^1^	No serious inconsistency	No serious indirectness	No serious imprecision	Reporting bias^2^	145	72	—	MD 0 (−0.58 to −0.01)	*⨁⨁*○○ Low	Important^3^

Psychotism (measured with: psychotism score of Symptom Checklist 90 at end of treatment; range of scores: 0–40; better indicated by less)												

3	Randomised trial	Serious^1^	No serious inconsistency	No serious indirectness	No serious imprecision	Reporting bias^2^	145	72	—	MD 0 (−0.83 to −0.24)	*⨁⨁*○○ Low	Important^3^

Paranoid ideation (measured with: paranoid ideation score of Symptom Checklist 90 at end of treatment; range of scores: 0–24; better indicated by less)												

3	Randomised trial	Serious^1^	No serious inconsistency	No serious indirectness	No serious imprecision	Reporting bias^2^	145	72	—	MD 0 (−0.62 to −0.05)	*⨁⨁*○○ Low	Important^3^

Psychological health (measured with: psychological score of Diabetes Specific Quality of Life Scale at end of treatment; range of scores: 0–40; better indicated by less)												

3	Randomised trial	Serious^4^	No serious inconsistency	No serious indirectness	No serious imprecision	Reporting bias^5^	120	52	—	MD 0 (−0.91 to −0.25)	*⨁⨁*○○ Low	Important^3^

^1^Wang et al. 2008 [30]: lack of allocation concealment and blinding, and failure to adhere to intention to treat principle when indicated; Lin 2007 [32]: lack of blinding; Zhang 2008 [31]: lack of blinding and reporting of some outcomes and not others on the basis of the results, ^2^Only three small studies were included, ^3^Further research is very much needed, ^4^Lin 2007 [32]: lack of blinding; Zhang 2008 [31]: lack of blinding and reporting of some outcomes and not others on the basis of the results, ^5^Only two small studies were included.
